# Phenotypic Basis for Matrix Stiffness-Dependent Chemoresistance of Breast Cancer Cells to Doxorubicin

**DOI:** 10.3389/fonc.2018.00337

**Published:** 2018-09-05

**Authors:** M. Hunter Joyce, Carolyne Lu, Emily R. James, Rachel Hegab, Shane C. Allen, Laura J. Suggs, Amy Brock

**Affiliations:** ^1^Department of Biomedical Engineering, University of Texas at Austin, Austin, TX, United States; ^2^Department of Biomedical Engineering, Louisiana Tech University, Ruston, LA, United States; ^3^Institute for Cellular and Molecular Biology, University of Texas at Austin, Austin, TX, United States

**Keywords:** chemotherapy, resistance, extracellular matrix, tumor microenvironment, 3D cell culture

## Abstract

The persistence of drug resistant cell populations following chemotherapeutic treatment is a significant challenge in the clinical management of cancer. Resistant subpopulations arise via both cell intrinsic and extrinsic mechanisms. Extrinsic factors in the microenvironment, including neighboring cells, glycosaminoglycans, and fibrous proteins impact therapy response. Elevated levels of extracellular fibrous proteins are associated with tumor progression and cause the surrounding tissue to stiffen through changes in structure and composition of the extracellular matrix (ECM). We sought to determine how this progressively stiffening microenvironment affects the sensitivity of breast cancer cells to chemotherapeutic treatment. MDA-MB-231 triple negative breast carcinoma cells cultured in a 3D alginate-based hydrogel system displayed a stiffness-dependent response to the chemotherapeutic doxorubicin. MCF7 breast carcinoma cells cultured in the same conditions did not exhibit this stiffness-dependent resistance to the drug. This differential therapeutic response was coordinated with nuclear translocation of YAP, a marker of mesenchymal differentiation. The stiffness-dependent response was lost when cells were transferred from 3D to monolayer cultures, suggesting that endpoint ECM conditions largely govern the response to doxorubicin. To further examine this response, we utilized a platform capable of dynamic ECM stiffness modulation to allow for a change in matrix stiffness over time. We found that MDA-MB-231 cells have a stiffness-dependent resistance to doxorubicin and that duration of exposure to ECM stiffness is sufficient to modulate this response. These results indicate the need for additional tools to integrate mechanical stiffness with therapeutic response and inform decisions for more effective use of chemotherapeutics in the clinic.

## Introduction

Cancer is a complex disease capable of affecting multiple properties of tissue organization and is driven by numerous factors. Hanahan and Weinberg (1, 2) have summarized and defined “hallmarks of cancer” which describe biological conditions characteristic of tumorigenesis. Pickup (3) revisited these hallmarks and described roles that the extracellular matrix (ECM) plays in each. The ECM includes the non-cellular components of a cell's microenvironment responsible for providing physical scaffolding and facilitating signaling from the surrounding tissue (4). Studies aimed at characterizing the role that physical cues play in tumor development have demonstrated that ligand type, ligand density, substrate composition, and substrate stiffness are important factors in tumor initiation, progression, and metastasis (5–12). Additional studies investigate how cells manipulate the ECM and vice-versa, suggesting a reciprocal nature whereby each shapes the response of the other (7, 8, 13–15).

With cellular remodeling of the ECM, tumors become progressively more rigid than surrounding tissue, a characteristic that informs diagnosis by physical palpitation and imaging (16). These serve as early detection methods for breast cancer (16), the second leading cause of cancer death among women (17). The ECM influences organization (15) and differentiation (18, 19) of mammary cells into functional mammary structures and tissues. It plays a role in tumor suppression through trafficking of intercellular signals (20) and exerting physical forces sensed by cell surface proteins, such as integrins (10, 13, 15). Changes in the ECM composition (15, 18, 19, 21) and stiffness (6–8, 15, 22, 23) have been shown to promote malignant phenotypes. Previous work in the field has investigated the role of ECM in determining cellular response to chemotherapy treatment (9, 24). Rice et al. (25) and Zustiak et al. (26) both demonstrate that ECM stiffness can alter chemotherapeutic response for some cancer cell lines. Shin and Mooney (9) examined the relationship between ECM ligands and cancer cell resistance to chemotherapeutics. Here we utilize a hydrogel platform to examine how ECM stiffness and the dynamic modulation of that microenvironmental stiffness affect the response of two distinct breast carcinoma cell lines to chemotherapeutic treatment.

Hydrogels have served to isolate and model aspects of the ECM for *in vitro* studies of cellular organization (15) and differentiation (18, 26, 27), tumor suppression (10, 13, 15), tumorigenesis (6, 13, 28–30), promotion of malignant phenotypes (6–8, 15, 22, 23), and cellular response to chemotherapy (9, 24, 31). Materials native to mammalian cellular ECM (i.e., scaffolding proteins such as collagens and fibrins) as well as biomimetic materials (such as agar and acrylamide) are used to produce 3D scaffolds for *in vitro* cultures. The mechanical properties of these hydrogel scaffolds are largely determined during the initial preparation; overall stiffness of the hydrogel is determined by the density of protein/polymer or number of cross-links formed. However, with these systems is it not possible to adjust matrix stiffness in a controlled manner after initial formation of the hydrogel. Recent work has shown that the contractile force of MDA-MB-231 cells can cause significant increases in stiffness of their surrounding ECM (32). Microrheology with optical tweezers has been used to measure the stiffness gradient created by cells grown in collagen hydrogels; the linear stiffness of hydrogels adjacent to MDA-MB-231 cells was measured to be up to two orders of magnitude stiffer than areas of unoccupied hydrogel 200 μm away. Similar stiffening was seen for MDA-MB-231 cells in Matrigel cultures and human umbilical vein endothelial cells in fibrin hydrogels, indicating that maintaining hydrogel stiffness within a defined range will be challenging as cells rearrange their ECM over time.

Our studies utilized the alginate-based hydrogel system described by Stowers et al. (33) as a means to predictably modulate hydrogel stiffness. Alginate is bio-inert, and alginate polymers can be cross-linked with calcium ions, providing a means for controlling hydrogel stiffness. Additionally, this system incorporates 1,2-dipalmitoyl-sn-glycero-3-phosphocholine (DPPC) liposomes loaded with calcium and gold nanorods. Irradiation with NIR light causes the gold nanorods to undergo surface plasmon resonance and heat the liposomes. As the liposomes approach the phase transition temperature (41°C), they undergo gel-to-liquid transition causing the encapsulated calcium to leak out and form additional alginate cross-links. This experimental platform allowed us to seed breast cancer cells into hydrogels of an initial stiffness (200 and 2,000 Pa), further stiffen their ECM during the course of the experiment (200 1,600 Pa and 2,000 3,000 Pa), and treat samples with the chemotherapeutic doxorubicin to determine how dynamic changes to the ECM affect chemotherapeutic resistance. Here we show that stiffness of the ECM is sufficient to modulate MDA-MB-231 breast cancer cell resistance to doxorubicin.

## Materials and methods

### Cell culture

MDA-MB-231 cells were cultured in Dulbecco's Modified Eagle's Medium (DMEM, Life Technologies, REF: 10569-010, 94%) supplemented with FBS (Life Technologies, REF: 10437-028 5%) and P/S (Life Technologies, REF: 15070-063, 1%). MCF7 cells were cultured in Minimum Essential Media (MEM, Life Technologies, REF: 11095-080, 89%) supplemented with Fetal Bovine Serum (FBS, Life Technologies, REF: 10437-028, 10%) and Penicillin/Streptomycin (P/S, Life Technologies, REF: 15070-063, 1%). All cultures were incubated at 37°C with 5% CO_2_.

### Hydrogel preparation

Hydrogels were prepared by mixing the following ingredients in the order described with thorough mixing after addition of each ingredient: 4% alginate (Pronova UP MVG; 40% total volume, 1.6% final concentration), 5–20 mM calcium carbonate (5% total volume), liposomes loaded 500 mM calcium chloride plus AuNRs (20% total volume), cells (8 million cells/mL; 5% total volume), 10–40 mM D-(+)-Gluconic acid δ-lactone (Sigma-Aldrich, G4750; 5% total volume), and Matrigel (VWR International, 47743-715; 25% total volume). Once mixed, 50 μL of the gel solution was pipetted into each well of a 96-well plate and placed in an incubator (37°C, 5% CO_2_) for 1 h to promote gelation. After gelation, 100 μL of media was added to each sample and placed back in an incubator.

### Liposome preparation

Liposomes were prepared using the interdigitation-fusion method described by Ahl et al. (34). 1,2-dipalmitoyl-sn-glycero-3-phosphocholine (DPPC, Avanti, 850355P/C) was diluted in chloroform (Fisher Scientific, CAS: 67-66-3) at 25 mg/mL and rotary evaporation (150 mbar vacuum, ~60 rpm, 55°C, 15 min.) was used to coat a round-bottom flask with thin layers of lipids. After 15 min. on the rotovap, the newly formed lipid cake was placed in a desiccator overnight to ensure complete evaporation of any residual chloroform. The lipid cake was rehydrated with 2 mL of ultra-pure water and placed on a rotator for 30 min. to ensure hydration of the entire lipid cake. The solution was then sonicated via sonic probe (60% amplitude for 10 min.) to form small unilaminar vesicles. At this point, the lipid solution was passed through a 0.22 μM filter (Millipore, SLGS033SB) and 424 μL of 100% ethanol (Fisher Scientific, CAS: 64-17-5) was added to form interdigitated sheets. Gold nanorods and 500 mM calcium chloride (Sigma-Aldrich, C1016) was added and allowed to incubate at 55°C for 2 h with gentle agitation every 30 min. to ensure encapsulation of cargo into newly forming liposomes. After incubation, a series of washes with 300 mM sodium chloride (Fisher Scientific, 7647-14-5) plus 1 mM HEPES (Sigma-Aldrich, H3375) was done to remove any free-floating nanorods or calcium.

### Dynamic stiffening of alginate hydrogels

Prior to exposing samples to near infrared light, all media was removed from each sample to minimize light scattering. A Lasermate (IML808-2500FLAM4A) set at 2.0 W was used to irradiate samples for 45 s each. After irradiating the final sample, 100 μL of fresh culture media was added to each well and the samples were placed back in an incubator (37°C, 5% CO_2_).

### Rheometry

To determine stiffness of the hydrogels used, gel solutions were prepared and pipetted into PDMS molds, incubated at room temperature for 2 h, and measured on a Physica MCR 101 Rheometer using an 8 mm geometry (Anton Paar, Cat.#: 5681). Rheoplus (v3.40) software was used to take frequency sweep measurements from 0.05 to 500 rad/s with 5% initial strain.

### Dosing with chemotherapeutics

Samples were exposed to doxorubicin (Sigma-Aldrich, Cat.#: D1515) for 48 h. A broad range (1 nM – 200 μM) of doses was used to determine the drug sensitivity.

### Isolation of cells from hydrogels

To extract the cells cultured in hydrogels for analysis, each 50 μL gel was soaked in 100 μL of 50 mM sodium citrate (Fisher Scientific, Cat#: BP327) for 15 min. at room temperature. Gels were then mechanically disrupted by pipetting until the alginate dissolved to a liquid solution. Each sample was transferred to a microcentrifuge tube and centrifuged at 600 × g for 10 min. to pellet.

### Measuring viability

Viability measures were assessed using acridine orange/propidium iodide (AOPI, Nexcelom, Cat#: CS2-0106) stain. Samples treated with AOPI were pelleted, resuspended in AccuMAX (Innovative Cell Technologies, Cat.#: AM-105), and mixed 1:1 with AOPI stain. A Nexcelom Cellometer Vision was used to count live cells. The data gathered from cell viability assays was fit to a sigmoid function in Microsoft Excel and used to calculate drug sensitivity, as measured by LD50 value.

### Staining for EMT markers

Samples were fixed with a 15 min. exposure to 4% paraformaldehyde at room temperature. Blocking and permeabilization buffer (0.1% Triton X-100 and 1% bovine serum albumin [BSA] diluted in 1 × PBS) was added to each sample and incubated at room temperature for 1 h. Following this, samples were incubated with primary antibody against YAP (Santa Cruz, sc-101199) for 1 h at room temperature. Cell nuclei were stained with 300 nM DAPI (ThermoFisher, D1306) for 30 min. at room temperature before being imaged on a Zeiss confocal or EVOS epifluorescent microscope.

### Quantitative real-time PCR

Total RNA was extracted using the RNeasy Mini Kit (Qiagen, 74104, Hilden, Germany) and was reverse-transcribed using the High-Capacity cDNA Reverse Transcription Kit (Applied Biosystems, 4368814) according to the manufacturer's instructions. Quantitative PCR was performed using the PowerUp SYBR Green Master Mix (Applied Biosystems, A25743) with 20 ng cDNA input in 20 μl reaction volume run on a ViiA7 Real-Time PCR system (Applied Biosciences). GAPDH (glyceraldehyde 3-phosphate dehydrogenase) expression level was used for normalization as a housekeeping gene. The primers for GAPDH (HK-GAPDH) and CDH1 (VHPS-1738; E-Cadherin) were designed and synthesized by RealTimePrimers.com (www.realtimeprimers.com).

### Statistical analysis

A two-tailed Student's *t*-test assuming unequal variance was used to compare samples, and a *p*-value of less than 0.05 was used to determine statistical significance. Microsoft Excel Solver was used to fit cell viability data to a sigmoidal function to generate LD50 curves.

## Results

### MDA-MB-231 cells exhibit stiffness-dependent resistance to doxorubicin

**Figure 1:**
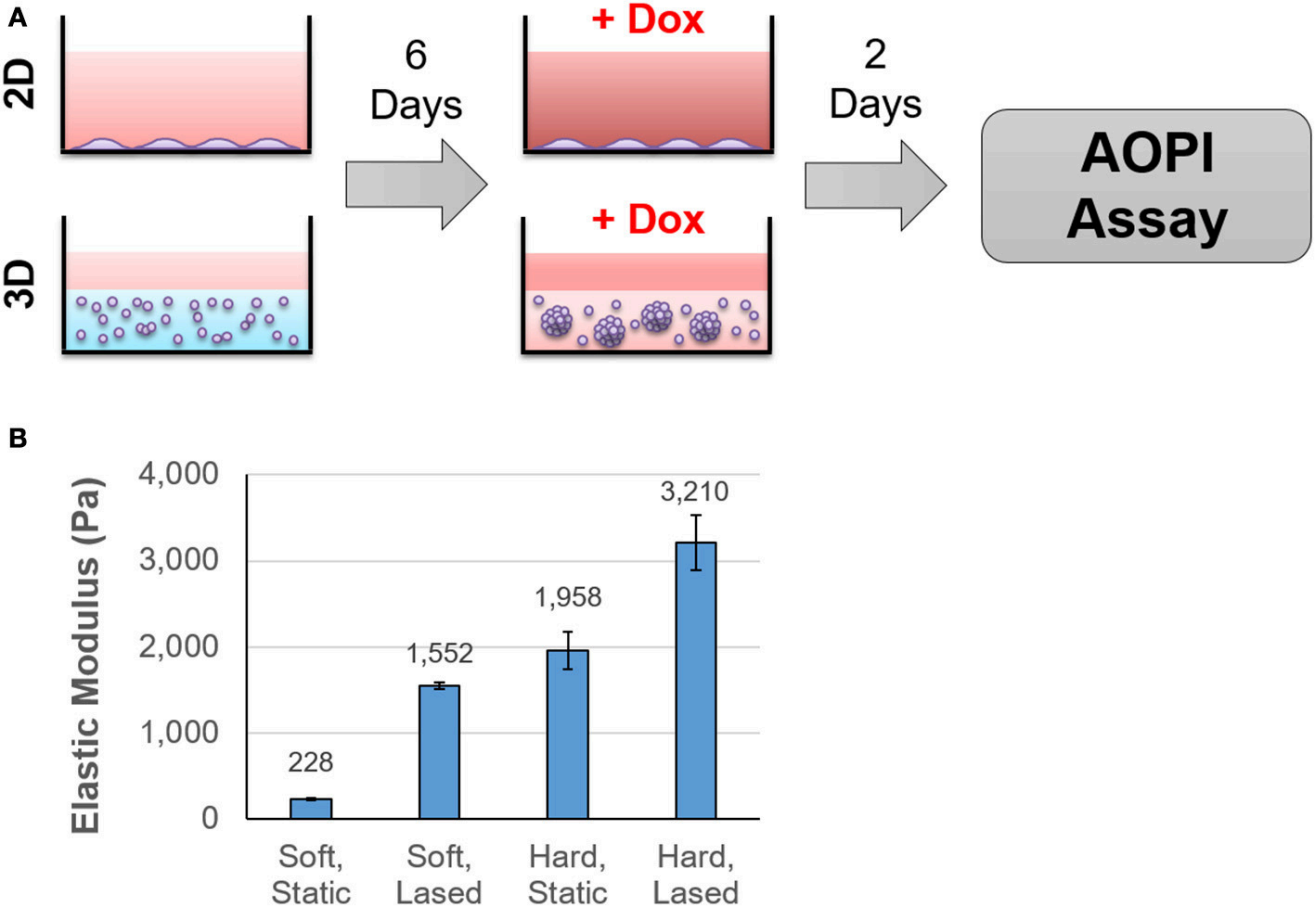
Cells were cultured in hydrogels of varying stiffness before treatment with doxorubicin. The experimental protocol is outlined in **(A)**. Briefly, cells were seeded onto tissue culture plastic or into hydrogels that ranged in stiffness from 200 to 2,000 Pa. After 6 days in culture, samples were exposed to doxorubicin for 48 h and cell viability was determined using AOPI staining. Hydrogel stiffness was determined by calculating Young's modulus from frequency sweep measurements obtained from a rheometer **(B)**.

**Figure 2:**
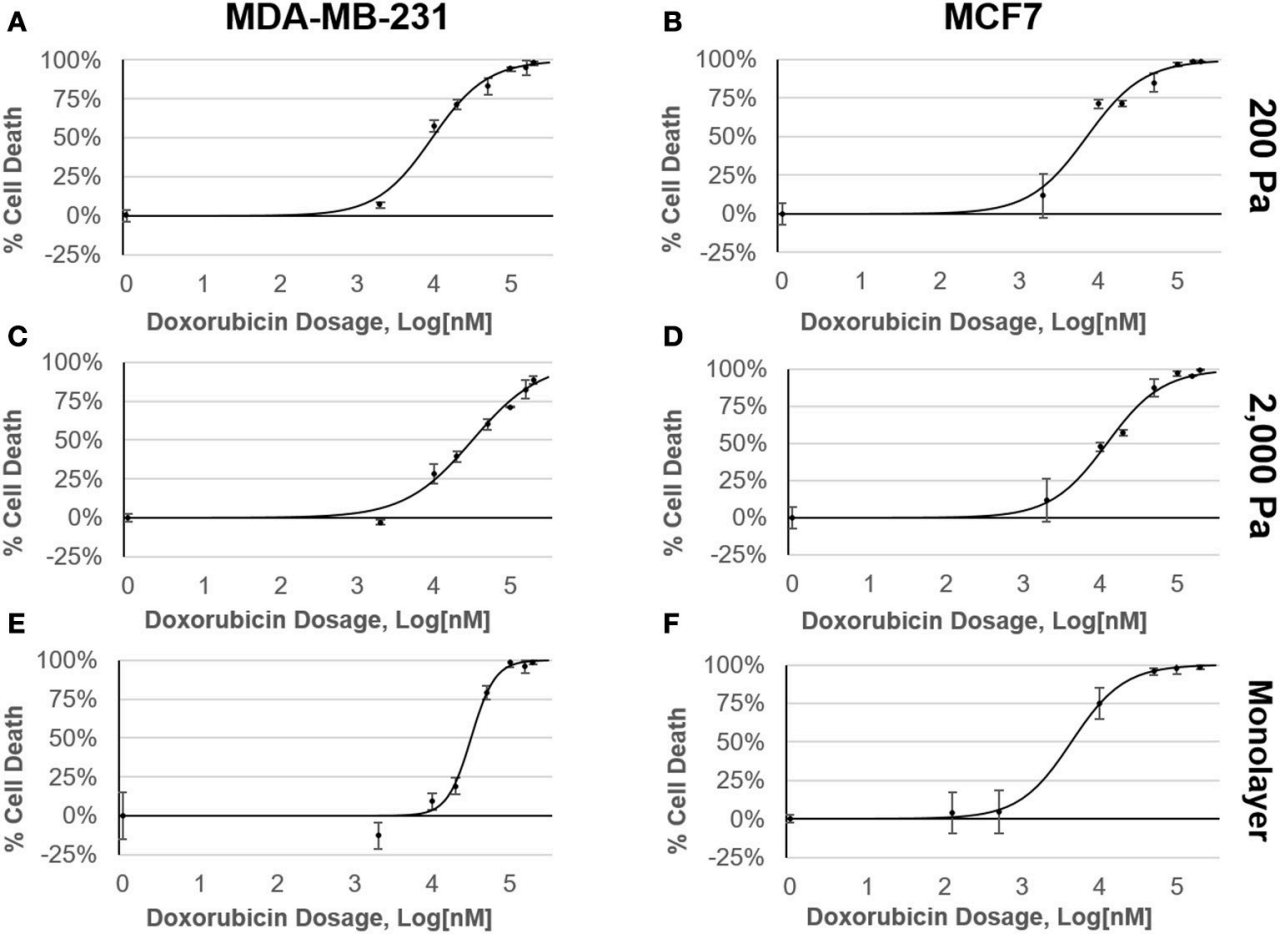
MDA-MB-231 cells have a stiffness-dependent resistance to doxorubicin. Dose response curves of MDA-MB-231 and MCF7 cells cultured in **(A,B)** 200 Pa hydrogel, **(C,D)** 2,000 Pa hydrogel, and **(E,F)** 2D monolayer following 48 h exposure to doxorubicin. Percent cell death was determined by staining samples with AOPI and counting live cells using a Nexcelom Cellometer.

Cells were cultured either as a monolayer on tissue culture plastic (2D) or in alginate-Matrigel hydrogels (3D) for six days before treatment with doxorubicin (Figure 1A). Oscillatory shear stress rheometry (1% strain, 0.5–50 Hz) was used to measure hydrogel stiffness. The elastic modulus was calculated for each frequency measured within this range (static soft = 228 Pa, *n* = 3; stiffened soft = 1,552, *n* = 2; static stiff = 1,958 Pa, *n* = 5; stiffened stiff = 3,210 Pa, *n* = 5; Figure 1B). We observed that chemoresistance of MDA-MB-231 cells to doxorubicin was 3-fold higher in the stiff ECM environment (LD50 = 10 μM in 200 Pa hydrogel cultures vs. LD50 = 32 μM in 2,000 Pa cultures; *p* = 0.002; Figures 2A,C). MCF7 cells did not display any significant (*p* = 0.134; Figures 2B,D) differences in resistance across substrates of increasing stiffness. When cultured as a monolayer on tissue culture plastic, MDA-MB-231 cells (LD50 = 27 μM; Figure 2E) were found to be more resistant (*p* = 0.001) to doxorubicin than similar MCF7 cultures (LD50 = 4 μM; Figure 2F).

**Figure 3:**
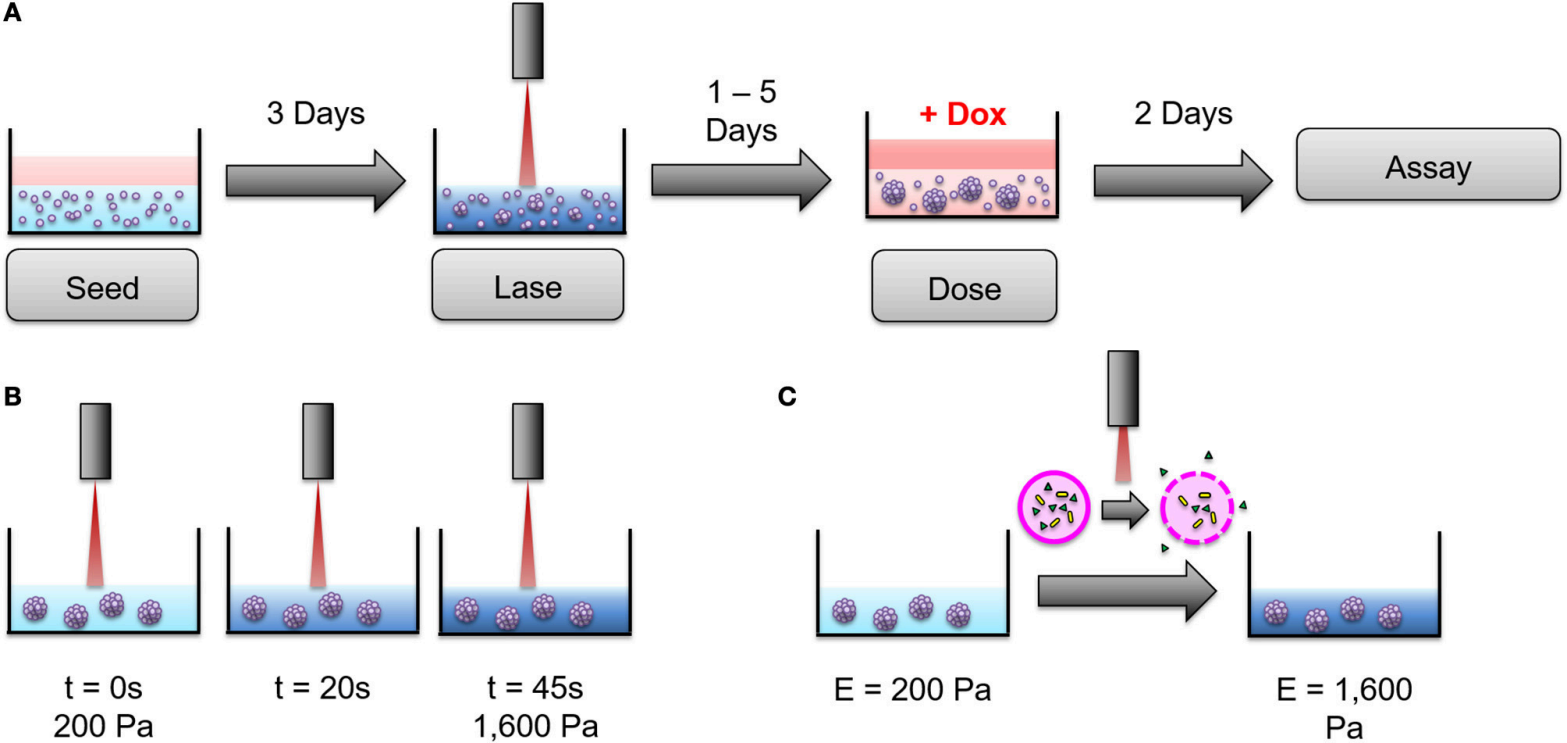
An alginate hydrogel platform was used to dynamically stiffen hydrogels to mimic progressive ECM stiffening. **(A)** Cells were seeded into hydrogels and cultured for 3 days before dynamic stiffening with NIR light. After stiffening, cultures were given 1–5 days to acclimate to the new stiffness of the hydrogel before being exposed to doxorubicin for 2 days (48 h). Following treatment with doxorubicin, viability assays were performed to determine doxorubicin resistance. **(B)** 200 Pa hydrogels were exposed to NIR light for 45 s to achieve ECM stiffness similar to 2,000 Pa static hydrogels. The same technique was used to stiffen 2,000 Pa hydrogels to 3,000 Pa. **(C)** NIR light induces surface plasmon resonance in encapsulated gold nanorods (gold) to heat liposomes (pink) close to their gel-to-liquid transition temperature. This causes calcium (green) to leak from the liposomes and form additional alginate cross-links, thereby stiffening the hydrogel. The above figure was adapted from Joyce et al. (35).

**Figure 4:**
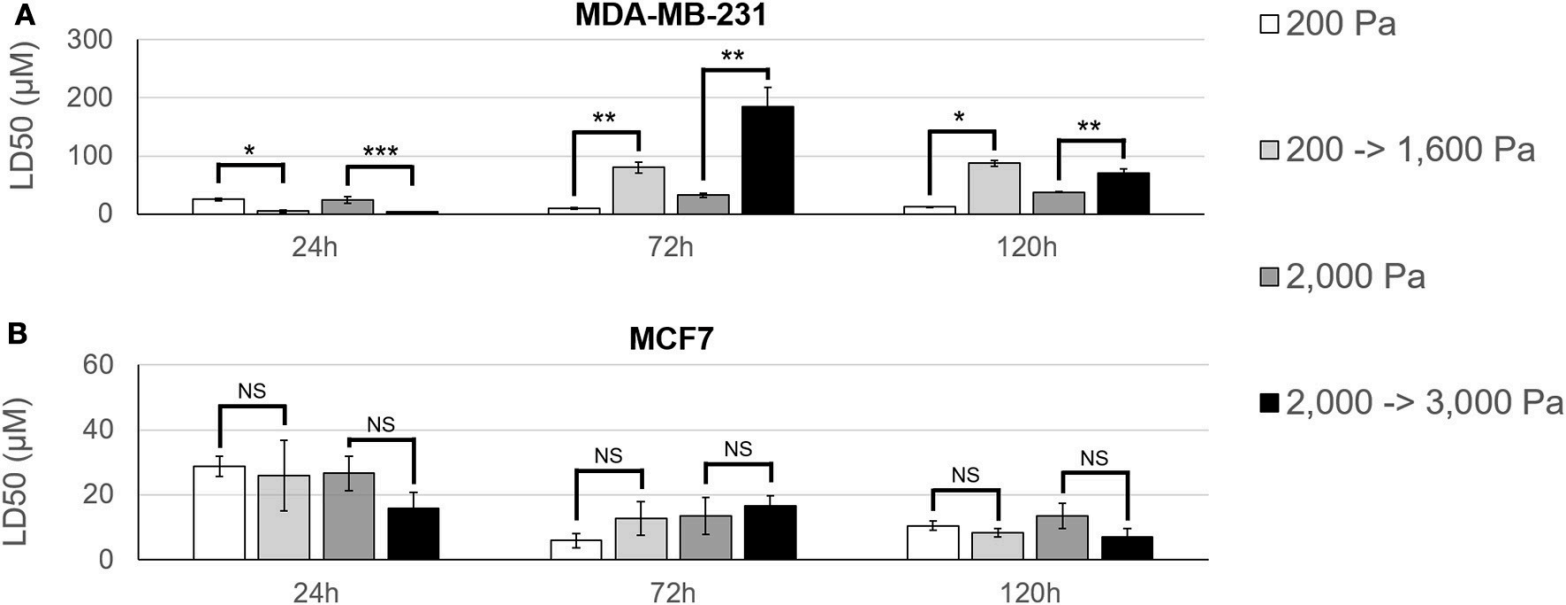
MDA-MB-231 cultures have an acclimation-dependent increase in resistance to doxorubicin. **(A)** MDA-MB-231 and **(B)** MCF7 cells were cultured in hydrogels with an initial stiffness of 200 Pa or 2,000 Pa for 3 days. On day 3, hydrogels either remained static (200 or 2,000 Pa) or were stiffened (200 –>1,600 Pa or 2,000 –>3,000 Pa) using NIR light. Samples were then given 24–120 h to acclimate to the hydrogel stiffness before 48 h treatment with doxorubicin. MDA-MB-231 cells showed a higher resistance to doxorubicin as hydrogel stiffness increased and this stiffness-dependent resistance was found to be partially dependent on duration of exposure to hydrogel stiffness. Comparable MCF7 samples did not show any significant change in resistance to doxorubicin across hydrogel stiffness or acclimation time. *p < 0.01; **p < 0.02; ***p < 0.03; NS, not significant, *p* > 0.05.

To further investigate this stiffness-dependence, cells were cultured in an alginate-Matrigel hydrogel platform, as described in Stowers et al. (33), which uses calcium-loaded liposomes to drive cross-linking following exposure to near infrared (NIR) light. Cells were initially cultured in hydrogels, for 3 days to begin formation of micro-structures (Figure 3A). Hydrogels were stiffened via NIR triggered cross-linking (Figures 3B,C), and cells were cultured for 3 additional days before treatment with doxorubicin for 48 h (Figure 3A). NIR light alone was not shown to significantly affect resistance to doxorubicin (*p* = 0.06; Supplementary Figure 1). MDA-MB-231 cultures that were grown in dynamically stiffened hydrogels had a significant decrease in sensitivity to doxorubicin for cultures that were stiffened from 200 Pa to 1,600 Pa (LD50 = 10 μM in 200 Pa static cultures vs. LD50 = 80 μM in cultures stiffened from 200 to 1,600 Pa; *p* = 0.004; Figure 4A) as well as those stiffened from 2,000 to 3,000 Pa (LD50 = 32 μM in 2,000 Pa static cultures vs. LD50 = 185 μM in cultures stiffened from 2,000 to 3,000 Pa, *p* = 0.014) compared to their static hydrogel counterparts (Figure 4A). This relationship was not observed in MCF7 (Figure 4B); there was no statistically significant difference in sensitivity to doxorubicin for cultures stiffened from 200 to 1,600 Pa (LD50 = 6 μM in 200 Pa static cultures vs. LD50 = 13 μM in cultures stiffened from 200 to 1,600 Pa; *p* = 0.143) or 2,000 to 3,000 Pa (LD50 = 12 μM in 2,000 Pa static cultures vs. LD50 = 17 μM in cultures stiffened from 2,000 to 3,000 Pa; *p* = 0.492) when compared to static cultures.

### Duration of acclimation modulates stiffness-dependent resistance

The time between dynamic stiffening of hydrogel cultures and treatment with doxorubicin was varied to determine if cells undergo adaptation to stiffened ECM that may alter drug sensitivity. Cells were cultured in hydrogels for 3 days to allow formation of micro-structures before being exposed to NIR to induce dynamic stiffening. Cultures were then maintained 24, 72, or 120 h in the stiffened ECM before treatment with doxorubicin. Resistance to doxorubicin peaked in MDA-MB-231 cells that were treated 72 h post-stiffening with an LD50 of 80 μM for cultures stiffened from 200 to 1,600 Pa and 185 μM for cultures stiffened from 2,000 to 3,000 Pa (Figure 4A). By 120 h post-stiffening, this increase in resistance dropped to 87 μM for cultures stiffened from 200 to 1,600 Pa (*p* = 0.323) and 71 μM for cultures stiffened from 2,000 to 3,000 Pa (*p* = 0.021). We hypothesize that the stiffening from 2,000 to 3,000 Pa elicits an acute response from MDA-MB-231 cells that results in an increased resistance to doxorubicin, which is shown at the 72 h acclimation time-point. However, our data suggests this response equilibrates by the 120 h acclimation time-point. Sensitivity of MDA-MB-231 to doxorubicin was greatest at 24 h post-stiffening with LD50 measures of 5 μM for cultures stiffened from 200 to 1,600 Pa and 3 μM for cultures stiffened from 2,000 to 3,000 Pa.

MCF7 cultures behaved inversely to their MDA-MB-231 counterparts (Figure 4B). Peak resistance for MCF7 cultures was seen at 24 h acclimation with an LD50 of 26 μM for cultures stiffened from 200 to 1,600 Pa and 16 μM for cultures stiffened from 2,000 to 3,000 Pa. Resistance progressively decreased to LD50 values of 13 μM (*p* = 0.154) and 8 μM (*p* = 0.104) for cultures stiffened from 200 to 1,600 Pa and 17 μM (*p* = 0.834) and 7 μM (*p* = 0.072) for cultures stiffened from 2,000 to 3,000 Pa at 72 and 120 h acclimation, respectively. When comparing across acclimation periods, however, these decreases in resistance were not statistically significant.

Dynamic stiffening had a significant difference for every acclimation duration tested with MDA-MB-231 cultures as determined by a Student's *t*-test with *p* < 0.05 denoting significance. This suggests that the duration of exposure to a particular set of ECM microenvironmental stiffness conditions contributes to resistance to doxorubicin in these cells.

### MDA-MB-231 increase markers of mesenchymal phenotypes on stiff ECM

**Figure 5:**
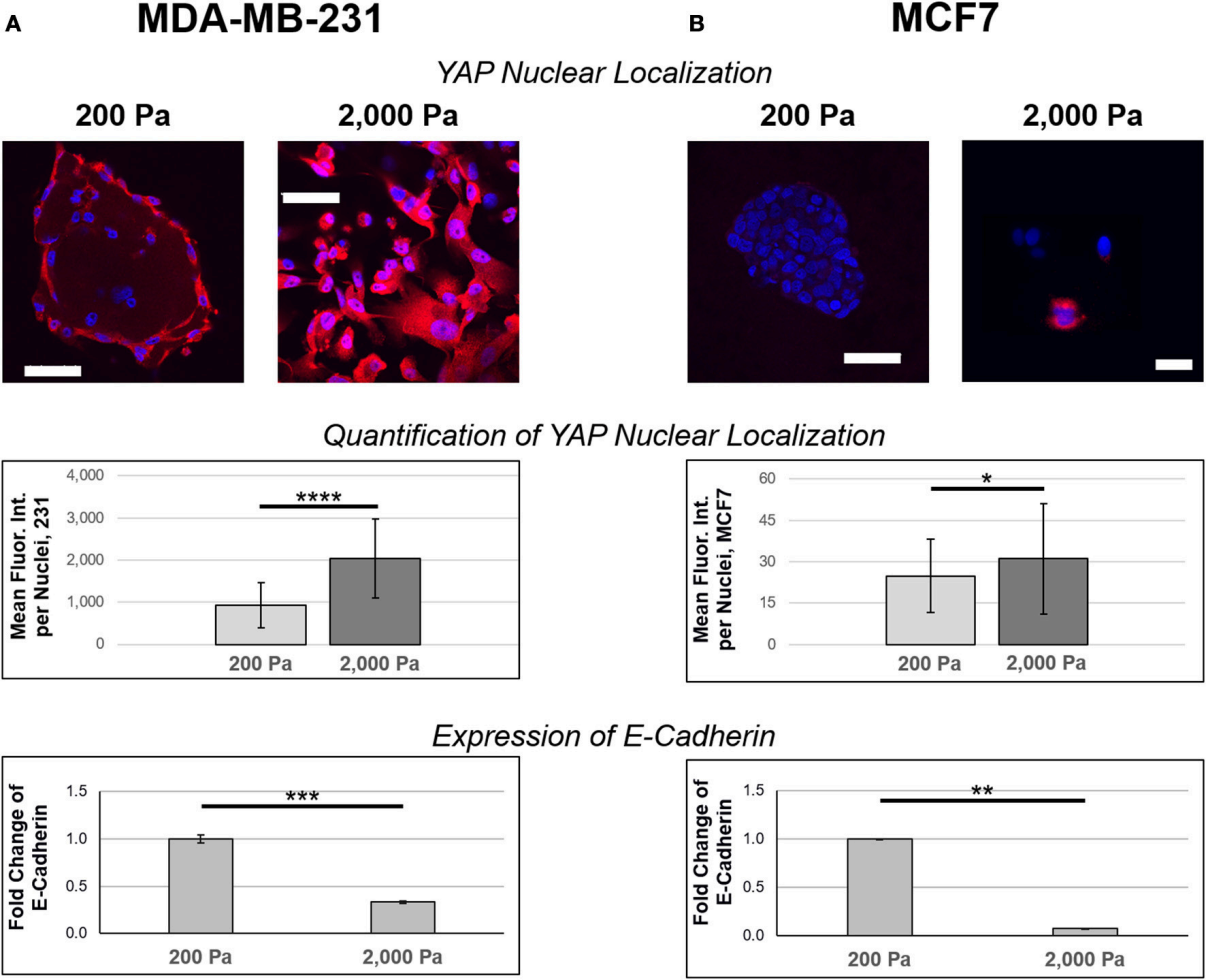
Stiffer ECM increases nuclear localization of YAP and decreases expression of E-Cadherin. **(A)** MDA-MB-231 and **(B)** MCF7 cells were cultured on 200 or 2,000 Pa hydrogels for 3 days before fixation and staining with YAP (red) antibodies and DAPI (blue). Images were captured using confocal microscopy and analysis was done in ImageJ to determine nuclear localization of YAP (*n* = 123 for MDA-MB-231 cells cultured in 200 Pa hydrogels, *n* = 119 for MDA-MB-231 cells cultured in 2,000 Pa hydrogels, *n* = 72 for MCF7 cells cultured in 200 Pa hydrogels, *n* = 90 for MCF7 cells cultured in 2,000 Pa hydrogels). There is a stiffness-dependent increase in nuclear localization of YAP for both 231 (*p* = 1.38E-22) and MCF7 (*p* = 0.02) cultures, though the increase in MCF7 cultures is small. MDA-MB-231 cultures showed significantly higher expression of nuclear YAP compared to similar MCF7 cultures (*p* = 2.37E-37 for 200 Pa and *p* = 4.44E-46 for 2,000 Pa). Quantitative PCR indicates that cells cultured in stiffer hydrogels have decreased expression of E-Cadherin for both MDA-MB-231 (*p* = 0.001) and MCF7 (*p* = 0.014) cultures. Scale bar = 50 μm. *p < 0.05; **p = 0.01; ***p = 0.001; ****p << 0.001.

Samples grown on 200 or 2,000 Pa hydrogels were also stained with YAP antibodies as a marker of mesenchymal phenotype. High levels of YAP nuclear localization confirmed that MDA-MB-231 cultures (Figure 5A) exhibited higher markers of mesenchymal phenotype than their MCF7 (Figure 5B) counterparts. The mean fluorescent intensity (MFI) of YAP-labeled nuclei was MFI = 930 a.u. in MDA-MB-231 cells cultured in 200 Pa hydrogels (*n* = 123) and MFI = 2,030 a.u. for the same cells cultured in 2000 Pa hydrogels (*n* = 119). Given the MDA-MB-231 cells grown on 2,000 Pa hydrogels showed higher levels of YAP nuclear localization than similar cultures on 200 Pa hydrogels (*p* = 1.36E-22), we believe that a stiffer ECM promotes the mesenchymal phenotype for these cells. Similar findings were observed in MCF7 cultures, but to a much lesser extent (*p* = 0.02) with MFI = 25 a.u. for cells cultured in 200 Pa hydrogels (*n* = 72) and MFI = 31 a.u. for cells cultured in 2,000 Pa hydrogels (*n* = 90). In addition, MCF7 cells cultured in 2,000 Pa hydrogels no longer grew in epithelial patches instead spreading more sparsely through the hydrogel suggesting a decreased propensity to bind to their neighbor cells. Quantitative real-time PCR showed a significant decrease in expression of E-Cadherin for both MDA-MB-231 (Figure 5A) and MCF7 (Figure 5B) cells cultured in 2,000 Pa hydrogels when compared to similar cultures in 200 Pa hydrogels. Decreased expression of E-Cadherin is a well-known marker of EMT, thus further supporting our findings that the stiffer hydrogels promote transition toward a mesenchymal phenotype.

## Discussion

In this current study, we sought to better understand how progressive stiffening of the ECM affects breast cancer response to chemotherapeutic treatment. Using an alginate-based hydrogel system, we observed that hydrogel stiffness and the duration of time that cells are exposed to ECM stiffness are sufficient to modulate resistance to doxorubicin in MDA-MB-231 cells. Hydrogels were prepared to mimic the stiffness of normal mammary tissue (200 Pa) and early stage breast tumors (2,000 Pa). When MDA-MB-231 cells were cultured in these conditions, doxorubicin resistance increased as the stiffness of the hydrogels increased. These findings were further verified when NIR light was used to dynamically stiffen cultures from normal mammary tissue stiffness (200 Pa) to approximately early stage breast tumor stiffness (1,600 Pa) and from early stage breast tumor stiffness (2,000 Pa) to a more advanced breast tumor stiffness (3,000 Pa). This stiffness-dependent resistance was not observed in similar MCF7 cultures, may be due to factors responsible for the phenotypic differences between the two cell lines.

Previous studies have demonstrated that epithelial-to-mesenchymal transition (EMT) is a critical factor in chemotherapeutic resistance across multiple cancer types including breast (36), cervical (37), ovarian (38), lung (39, 40), nasopharyngeal (41), and prostate (42). Rice et al. (25) were able to link ECM stiffness to induction of EMT in pancreatic cancer cells, which resulted in increased resistance to paclitaxel and gemcitabine. Shin and Mooney (9) found that stiffness of the microenvironment and presence of specific ligands was sufficient to increase myeloid leukemia resistance to select chemotherapeutics. These studies support our findings and highlight a role for EMT and microenvironment stiffness in chemotherapeutic resistance.

Yes-associated protein (YAP) is a transcriptional regulator that induces expression of proliferation and anti-apoptotic genes by shuttling between the cytoplasm and nucleus to interact with transcription factors (43). It is directly regulated by ECM and translocation to the nucleus is high in cells cultured on stiff ECM (44–47) which can then trigger EMT (48–51) and increased drug resistance (52). Here we used confocal microscopy to visualize and quantify YAP nuclear localization for both MDA-MB-231 and MCF7 cells grown on 200 and 2,000 Pa hydrogels. MDA-MB-231 cultures showed higher levels of YAP nuclear localization on the stiffer (2,000 Pa) hydrogels, this relationship was much weaker in MCF7 cultures. This data agrees with similar findings from the literature linking YAP nuclear localization to EMT and subsequent increases in drug resistance.

The hydrogel system used here allows dynamic control of stiffening though NIR release of calcium. Here additional stiffening occurs over the course of hours, though breast cancer cells would experience similar microenvironment stiffening over the course of months or years *in vivo*. Our study identifies the need for an *in vitro* system that could be used to modulate cell microenvironment stiffness over a longer time-frame to more accurately mimic the progressive stiffening breast cancer cells experience *in vivo*.

In conclusion, our work has found that MDA-MB-231 cells have a stiffness-dependent resistance to doxorubicin. The duration of exposure to ECM stiffness is sufficient to modulate this resistance, but MDA-MB-231 cells grown in stiffer hydrogels were more resistance to doxorubicin treatment for all acclimation durations tested after 24 h. MCF7 cultures did not show a stiffness-dependent resistance to doxorubicin, which may be due to factors underlying their epithelial phenotype. Nuclear localization of YAP increased with hydrogel stiffness in MDA-MB-231 but not MCF7 cultures, indicating hydrogel stiffness alone was not sufficient to induce EMT in MCF7 cells but was sufficient to increase expression of the mesenchymal phenotype in MDA-MB-231 cells. This work highlights the importance of microenvironment stiffness in studies of chemotherapeutic resistance and demonstrates that progressive stiffening of the microenvironment can modulate drug resistance.

## Author contributions

AB and MJ: planning of the study and writing of the manuscript; CL, EJ, MJ, RH, and SA: conducting of experiments; All authors: analysis of the manuscript.

### Conflict of interest statement

The authors declare that the research was conducted in the absence of any commercial or financial relationships that could be construed as a potential conflict of interest.

## Supplementary material

Supplementary Figure 1 Exposure to Near Infrared (NIR) light does not significantly affect response to doxorubicin. MDA-MB-231 cells were cultured as a monolayer (2D) on tissue culture plastic before being exposed to NIR light for 45 s. Samples were cultured an additional 2 days after lasing prior to treatment with doxorubicin. Staining with acridine orange/propidium iodide (AOPI) was used to quantify the ratio of live/dead cells. **(A)** Cells that were exposed to NIR light did not show a significant difference (*p* = 0.064) in resistance to doxorubicin compared to **(B)** control samples that were not exposed to NIR light. *n* = 3.
