# Supplementary figures and images for: Phenotypic Basis for Matrix Stiffness-Dependent Chemoresistance of Breast Cancer Cells to Doxorubicin
Source: Front Oncol. 2018 Sep 5;8:337. doi: 10.3389/fonc.2018.00337 (PMC6134055; doi:10.3389/fonc.2018.00337)

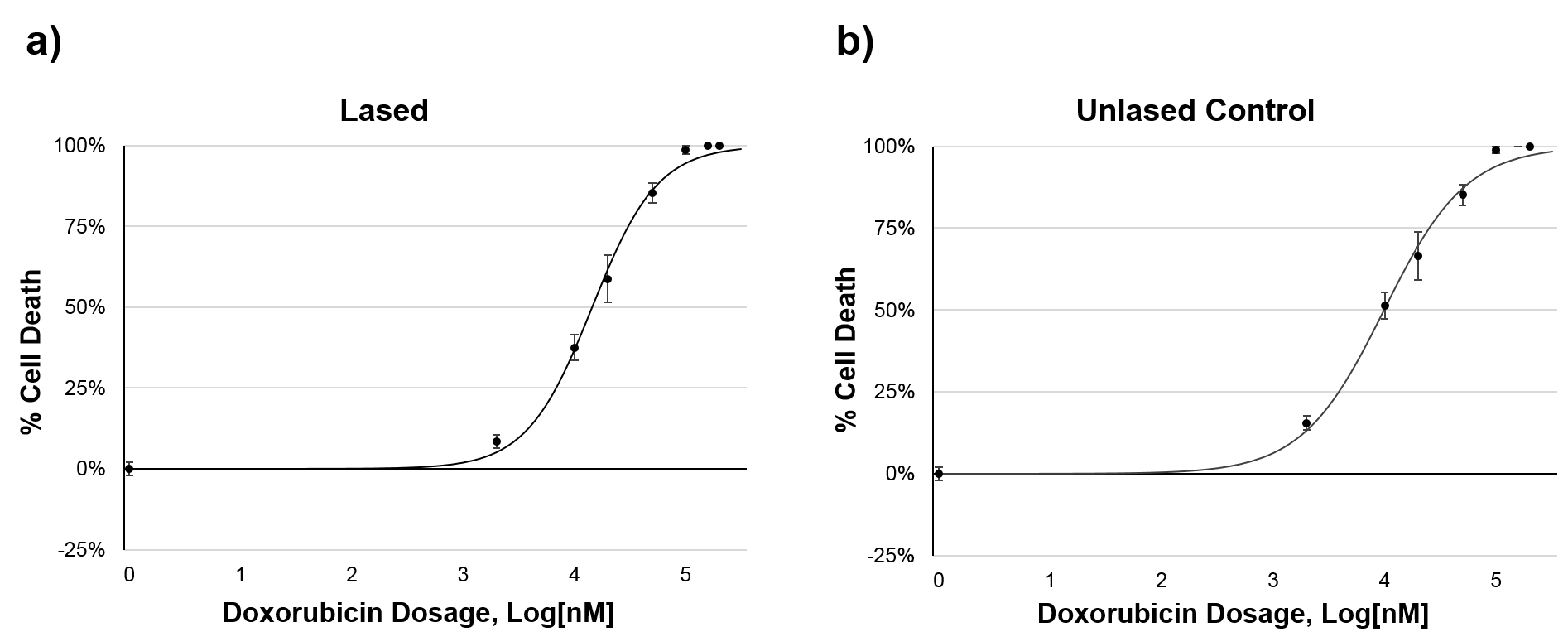

Supplement: Supplementary Figure 1 — Exposure to Near Infrared (NIR) light does not significantly affect response to doxorubicin. MDA-MB-231 cells were cultured as a monolayer (2D) on tissue culture plastic before being exposed to NIR light for 45 s. Samples were cultured an additional 2 days after lasing prior to treatment with doxorubicin. Staining with acridine orange/propidium iodide (AOPI) was used to quantify the ratio of live/dead cells. (A) Cells that were exposed to NIR light did not show a significant difference (p = 0.064) in resistance to doxorubicin compared to (B) control samples that were not exposed to NIR light. n = 3. [file Image_1.TIF]
